# Fluoroless Ablation of a Coronary Sinus–related Accessory Pathway Without Coronary Sinus Angiogram in a Patient with Multiple Prior Failed Ablations

**DOI:** 10.19102/icrm.2026.17091

**Published:** 2026-09-15

**Authors:** Anthony Costa, Fayaz Hakim, Jennifer Burchell, Megan Nettle, Khalil Kanjwal

**Affiliations:** 1 Department of Internal Medicine, Henry Ford Genesys Hospital, Grand Blanc, MI, USA; 2 Division of Cardiology, Clinical Cardiac Electrophysiology, Henry Ford Genesys Hospital, Grand Blanc, MI, USA; 3 Cardiac Cath Laboratory, Henry Ford Genesys Hospital, Grand Blanc, MI, USA

**Keywords:** 3D electroanatomic mapping, accessory pathway, coronary sinus venography, epicardial pathway, zero-fluoroscopy

## Abstract

Accessory pathways (APs) associated with the coronary sinus (CS), often linked to diverticula, are rare and challenging to ablate, frequently requiring CS venography for anatomical guidance. We report the case of a 56-year-old woman with recurrent palpitations, manifest pre-excitation, and two prior failed ablations for a presumed posteroseptal pathway. The electrocardiogram showed an immediately negative QRS complex in the inferior leads, suggesting an epicardial CS-related pathway. Using solely three-dimensional (3D) electroanatomic mapping (CARTO™; J&J MedTech, New Brunswick, NJ, USA) without fluoroscopy or CS angiogram, we reconstructed the geometry of the right atrium, venae cavae, and CS. Mapping revealed early ventricular activation and fused atrioventricular signals in distal CS, enabling successful radiofrequency ablation at 30 W with immediate loss of antegrade conduction. No retrograde conduction persisted post-adenosine, and tachycardia was non-inducible. The patient remained symptom-free at 1-year follow-up. To the best of our knowledge, this is the first reported successful fluoroless ablation of a CS-related epicardial AP without venography, highlighting the role of advanced 3D mapping in reducing radiation and contrast risks in complex cases.

## Introduction

Accessory pathways (APs) related to the coronary sinus (CS) are rare and can present with specific electrocardiographic features. The inability to identify classic electrocardiographic patterns and failure to map in the CS often result in failed ablation and recurrences. It is crucial to identify the electrocardiographic manifestations of such pathways for a better patient outcome. Usually, a CS venogram is often performed when these pathways are suspected, as identifying CS anatomy and the diverticulum can help localize the ablation target and improve ablation success. However, in this case report, we describe the successful use of three-dimensional (3D) electroanatomic mapping without fluoroscopy or CS venography to map and ablate a CS-related epicardial AP.

## Case presentation

A 56-year-old woman with a history of recurrent palpitations, documented narrow complex tachycardia, manifest pre-excitation on a 12-lead electrocardiogram (ECG), and two prior failed ablations in the right posteroseptal region was referred to our hospital for a second opinion. The patient was previously thought to have a posteroseptal AP; however, her ECG demonstrated an immediately negative QRS complex in the inferior leads suggestive of an epicardial CS diverticulum-associated pathway **(Figure 1)**.

**Figure 1:**
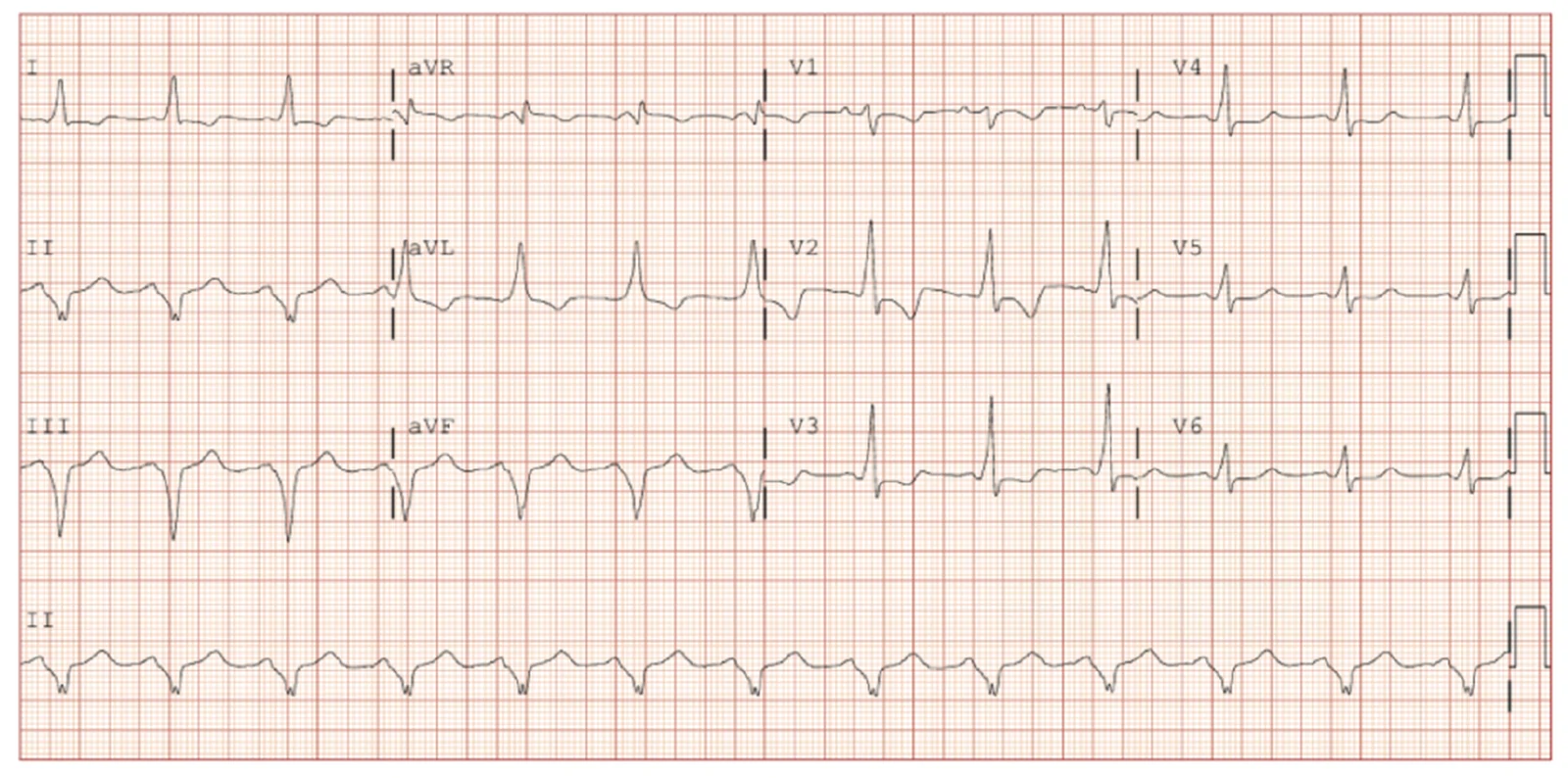
Twelve-lead electrocardiogram (ECG) demonstrating manifest pre-excitation. The ECG shows a short P–R interval with a delta wave and an immediately negative QRS complex in the inferior leads (II, III, and aVF), consistent with an epicardial accessory pathway.

Given her recurrent palpitations and two prior failed ablations, the patient was offered a repeat electrophysiology study. The risks and benefits of the procedure were discussed with the patient, and informed consent was obtained. During her previous ablations, mapping was performed in both the right and left posteroseptal regions but not within the CS. Venous access was obtained via bilateral femoral venous punctures, and a matrix was secured using a 3.5-mm irrigated-tip force-sensing ablation catheter. The CARTO™ mapping system (J&J Medtech, New Brunswick, NJ, USA) and a 3.5-mm irrigated-tip ablation catheter were used to create a 3D anatomical map of the right atrium (RA), superior vena cava, inferior vena cava, and CS **(Figure 2)**. The fast anatomical map of the CS and its proximal branch was reconstructed using an ablation catheter. The His-bundle region was also tagged. Electrophysiology catheters were placed in the high RA, right ventricular apex, His-bundle region, and CS. Baseline electrophysiological characteristics are shown in **Figures 1 and 3**. Notably, there was manifest pre-excitation and a short H–V interval. The patient was inducible for orthodromic reciprocating tachycardia (ORT) **(Figure 4)**. Attempts at ventricular entrainment of the tachycardia resulted in termination of the tachycardia; however, the first stable paced ventricular tachycardia entrained the tachycardia and was suggestive of ORT **(Figure 5)**. Antegrade mapping of the pathway was performed during an atrial pacing rhythm in the posteroseptal area of the tricuspid valve and within the CS. Using the guidance of the 3D reconstruction created, a 3.5-mm irrigated-tip ablation catheter was advanced into the proximal branch of the CS. A very early ventricular signal **(Figure 6)** and a fused atrioventricular (AV) signal were noted **(Figure 7)**. Post-ablation, the H–V interval increased to 45 ms (**Figure 8**), and a separated atrioventricular signal was noted at the ablation target site **(Figure 9)**. Ablation was performed in the power control mode at 30 W using an irrigated-tip catheter with an irrigation rate of 2 mL/min. The initial impedance noted before the ablation in the target area was 155 Ω, with an impedance drop of 12–15 Ω during energy delivery. Impedance, temperature, and catheter contact force were continuously monitored throughout ablation to optimize success and safety.

**Figure 2:**
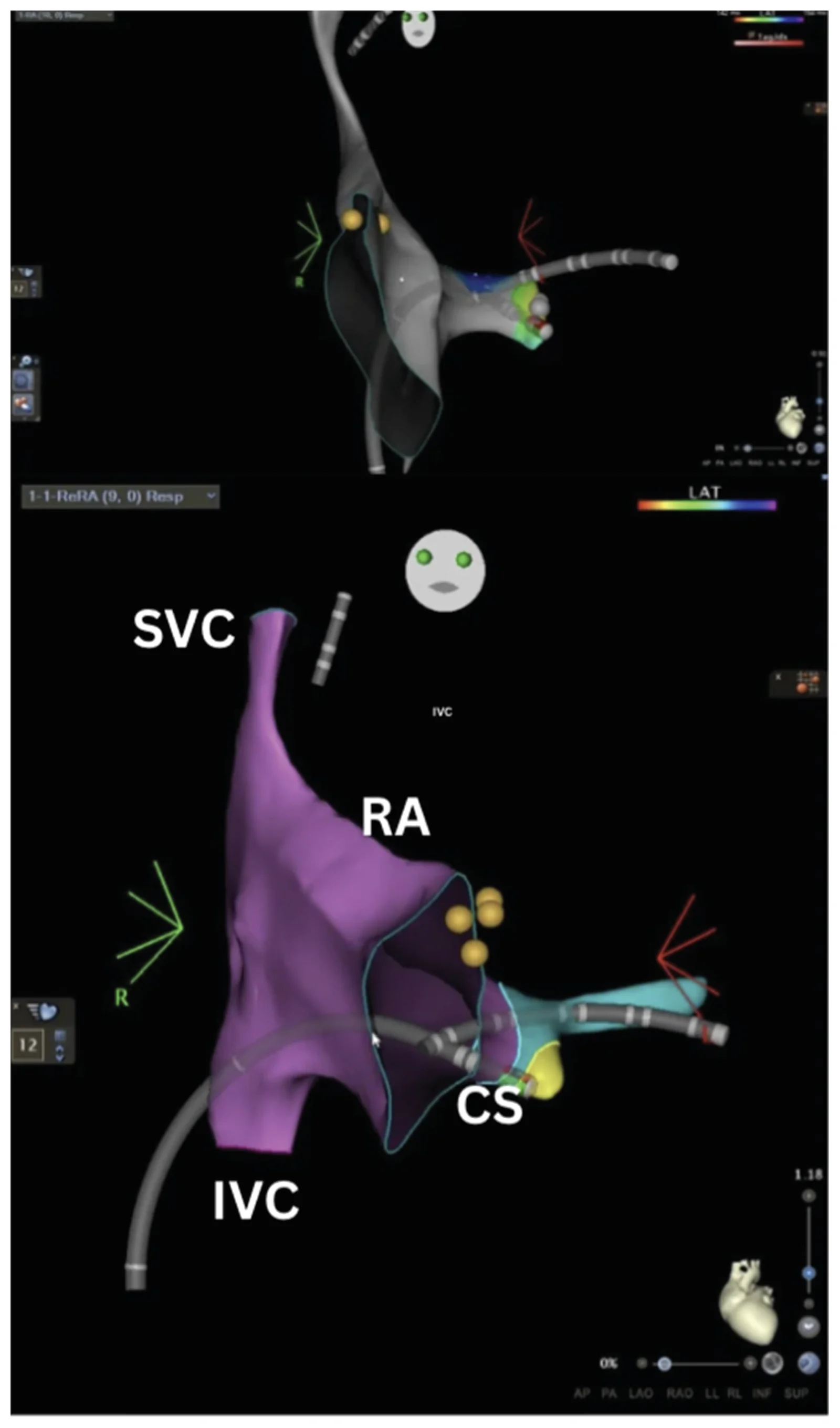
Three-dimensional electroanatomic map of the superior vena cava (SVC), inferior vena cava (IVC), right atrium (RA), and coronary sinus (CS) showing the ablation catheter at position in relation to the CS catheter.

**Figure 3:**
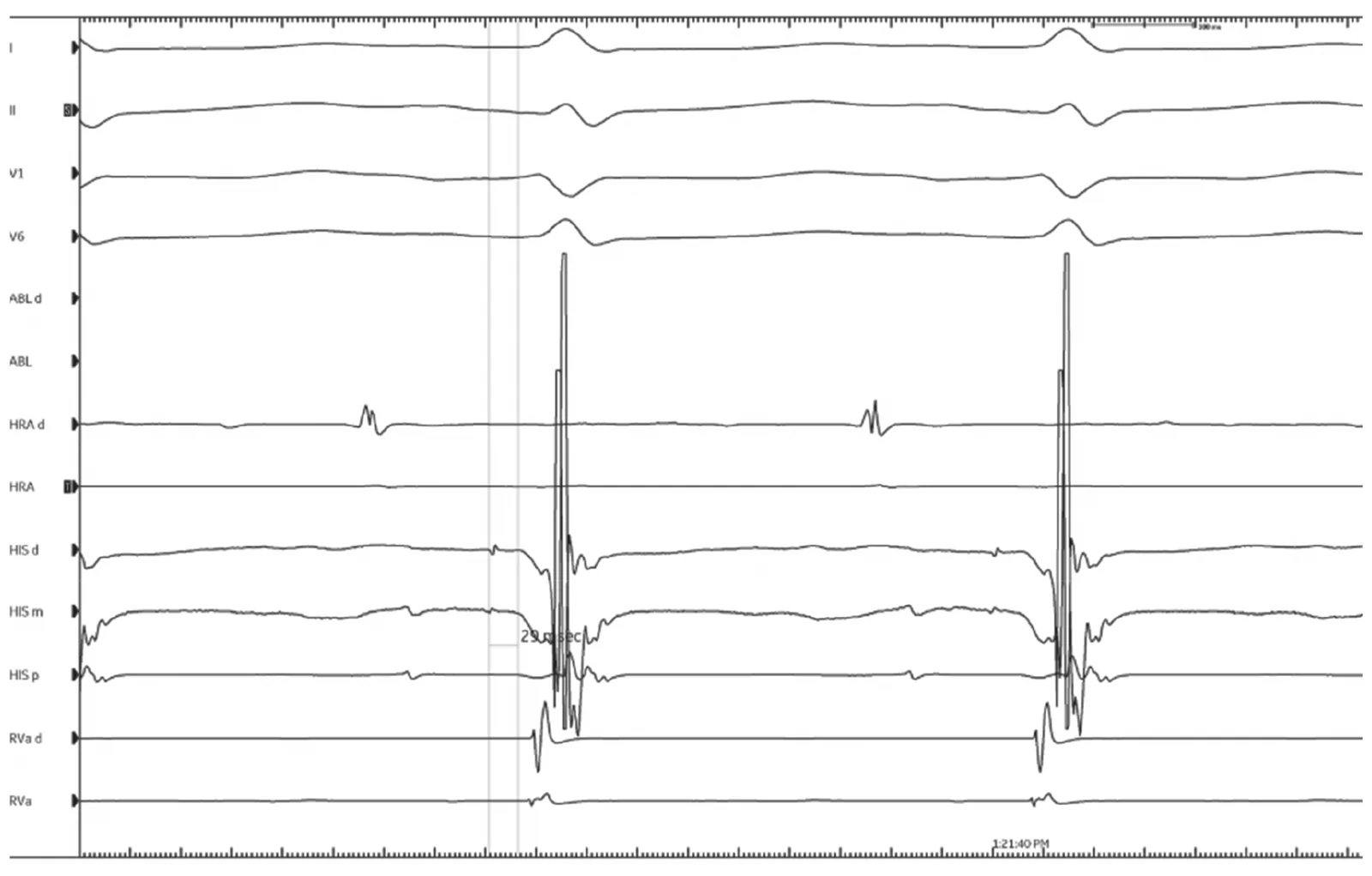
Intracardiac electrograms showing a short H–V interval of 29 ms.

**Figure 4:**
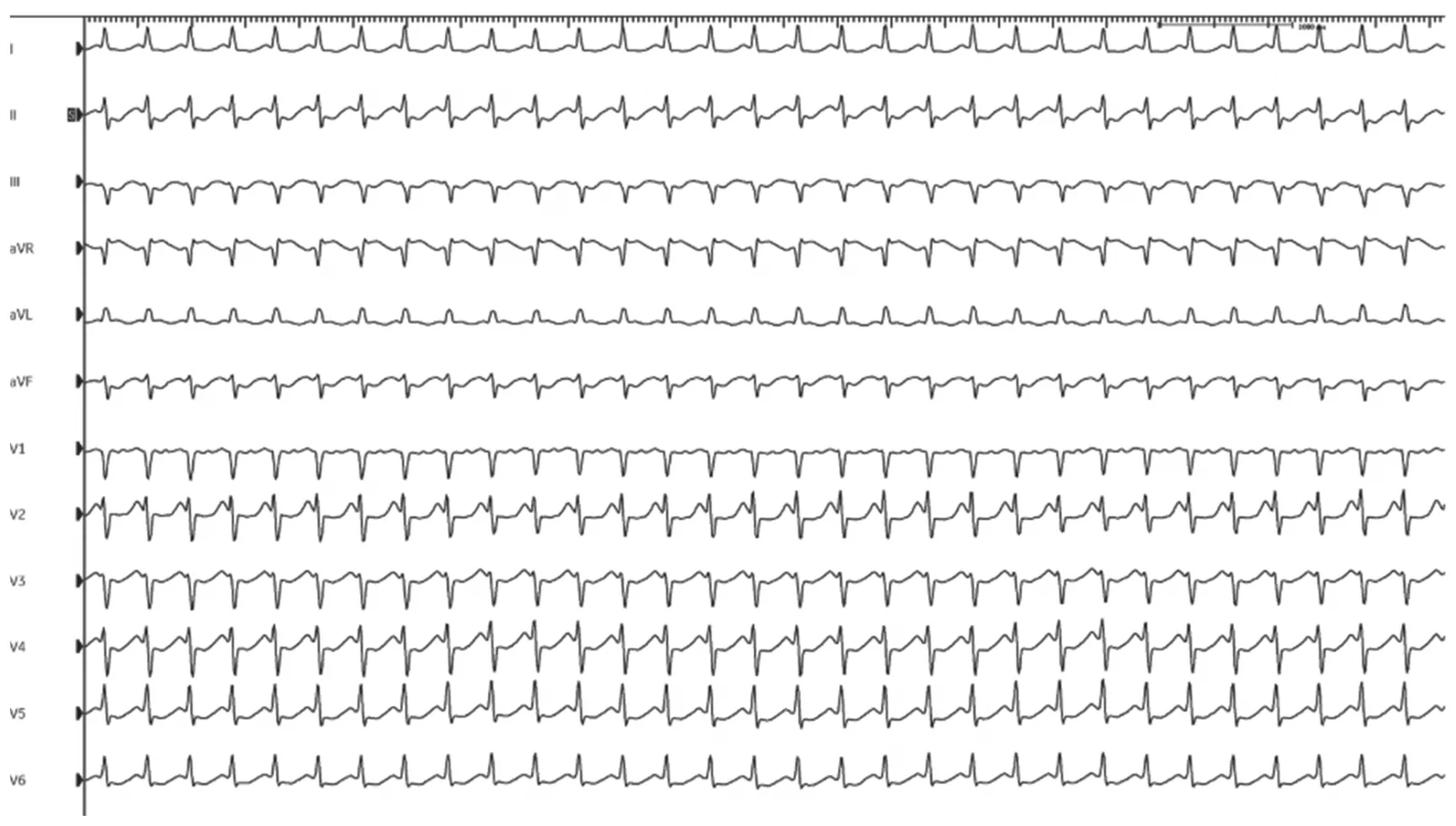
Narrow complex tachycardia inducible with a cycle length of 340 ms.

**Figure 5:**
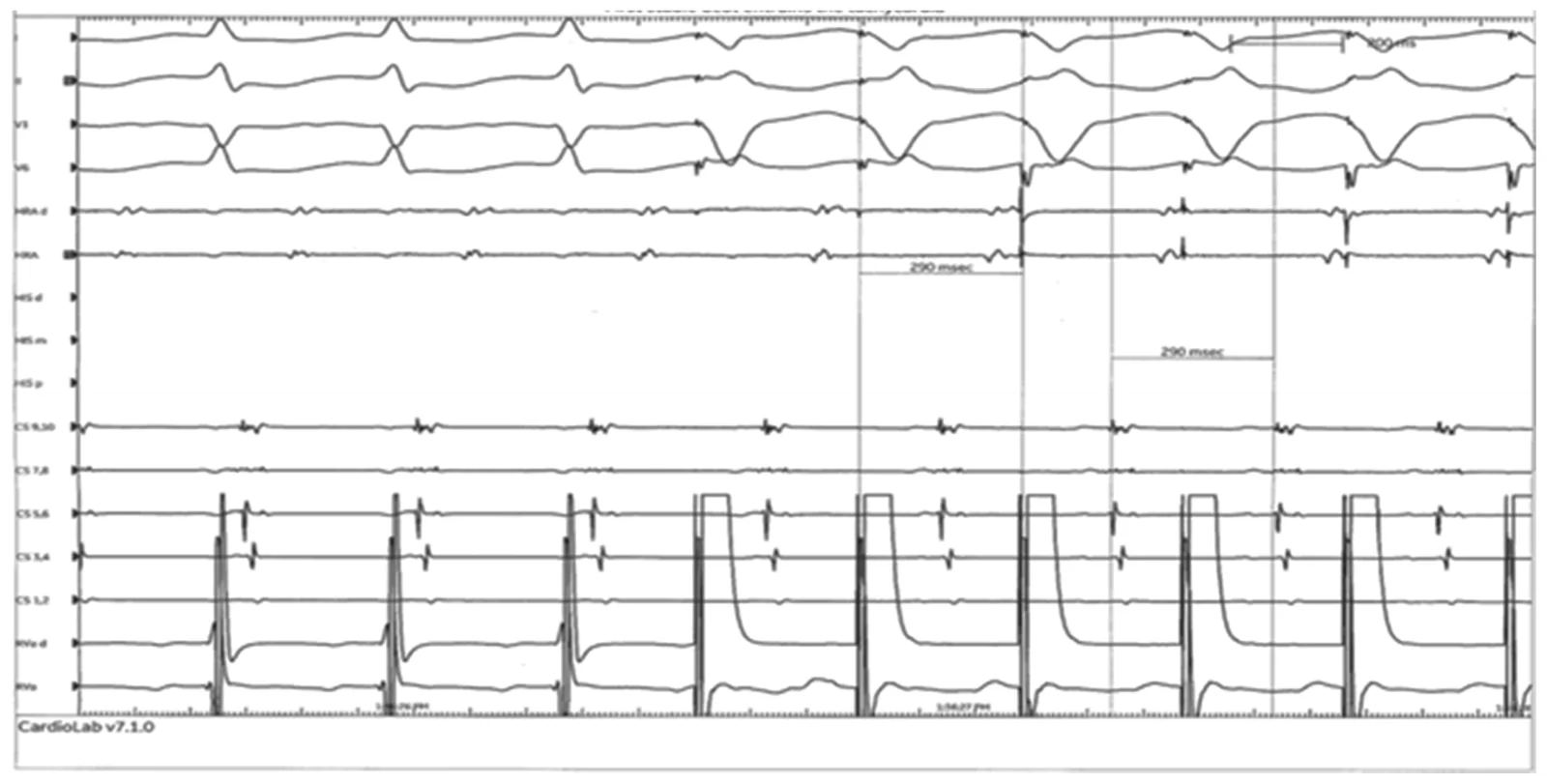
Ventricular entrainment of the tachycardia resulted in repeated termination of the tachycardia. However, the first stable ventricular beat entrained the tachycardia in a manner suggestive of orthodromic reciprocating tachycardia.

**Figure 6:**
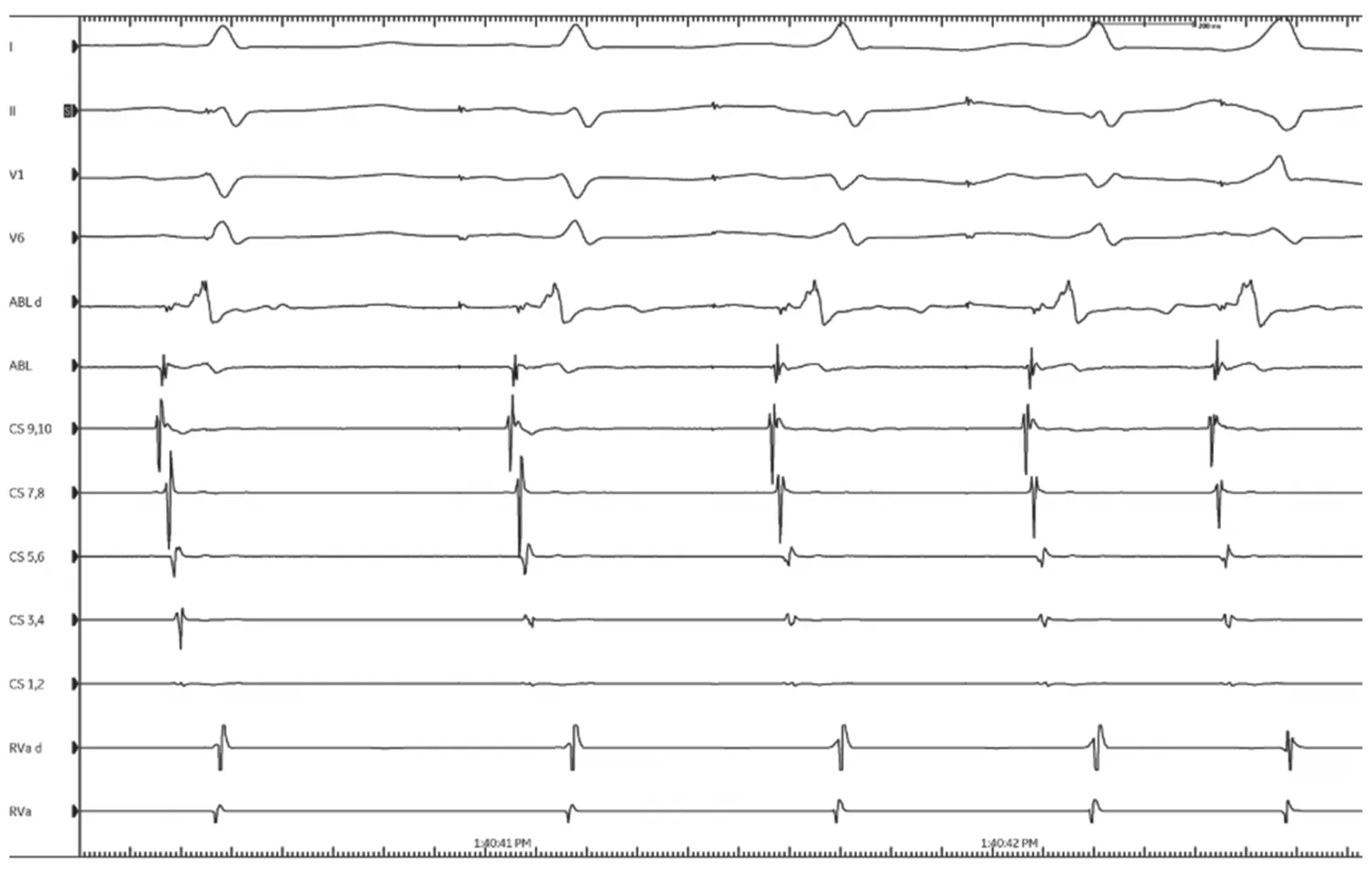
Ablation delivered at the site of the earliest ventricular signal recorded on the mapping system.

**Figure 7:**
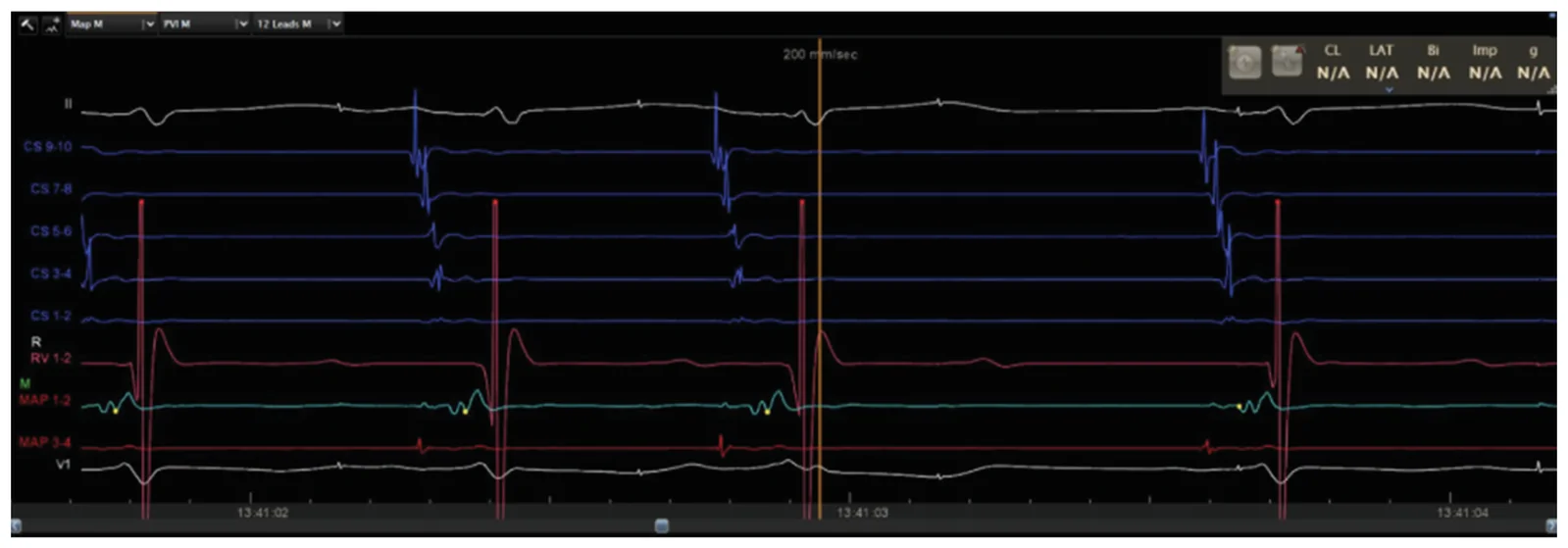
A fused local atrioventricular signal and a QS pattern noted on the unipolar electrode.

**Figure 8:**
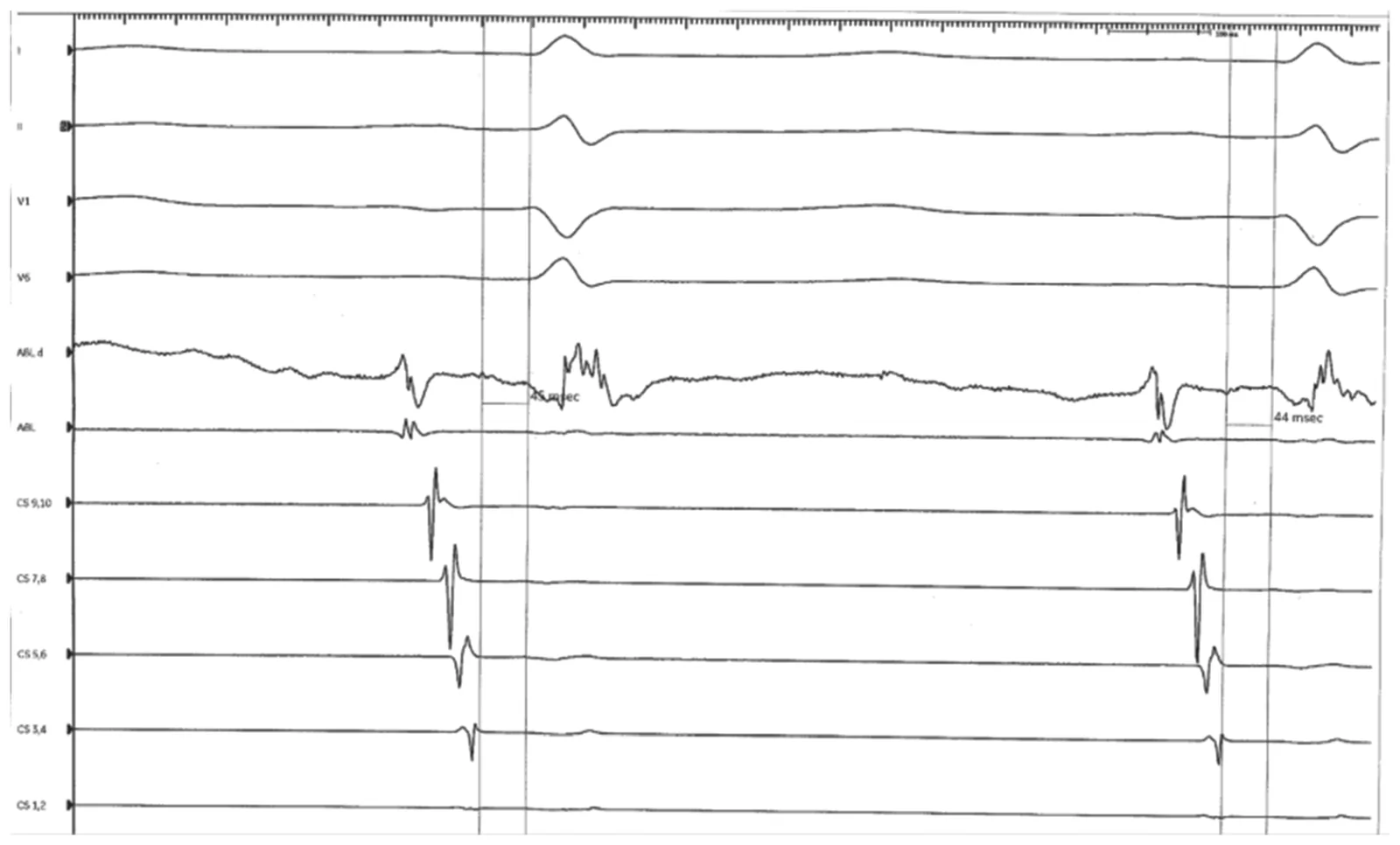
Post-ablation H–V interval of 45 ms.

**Figure 9:**
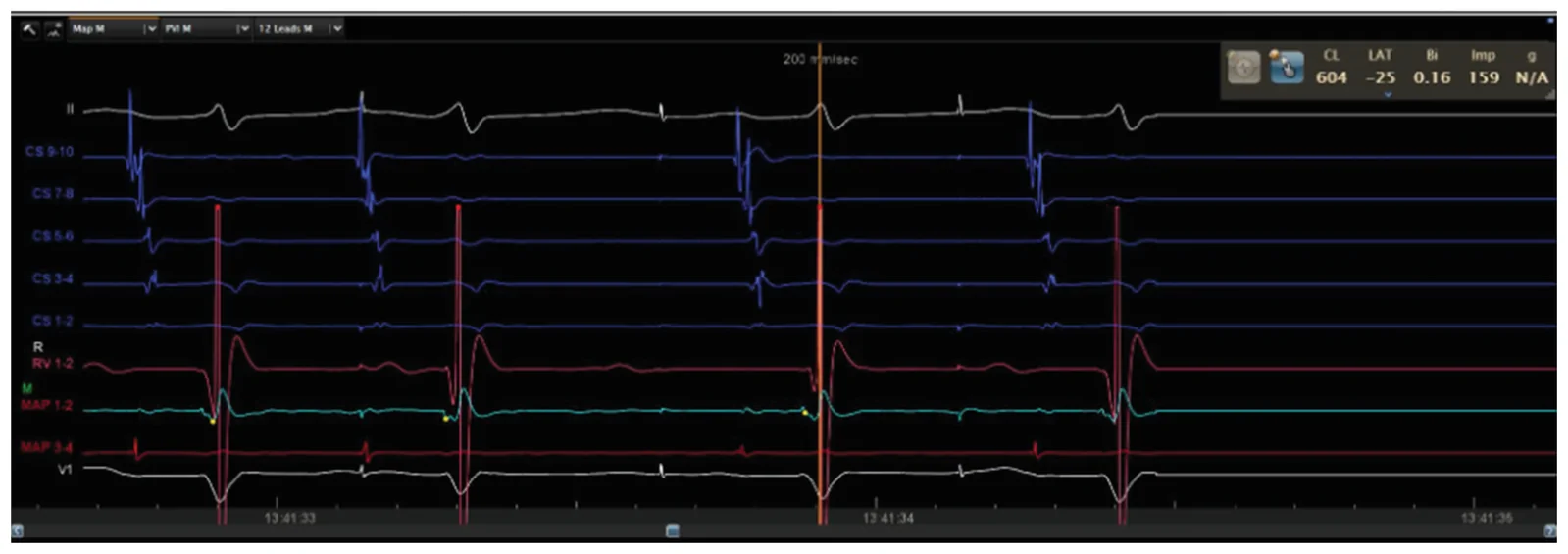
A separated atrioventricular signal noted on the ablation target site after ablation.

Ablation immediately abolished the antegrade conduction over the AP **(Figure 7)**. The ablation was continued for 60 s and, after a waiting period of 30 min, the antegrade pathway conduction did not return. Ventricular pacing was performed after administering 18 mg of adenosine intravenously, which showed no ventriculoatrial conduction, suggestive of no retrograde conduction pathway, and a post-ablation ECG showed loss of pre-excitation and loss of the delta wave, suggestive of antegrade conduction loss over the AP **(Figures 10A and 10B)**. Programmed electrical stimulation, both on and off isoproterenol, failed to induce any tachycardia. The patient was discharged home and was symptom-free at 6 months and 1 year of follow-up in our rhythm clinic.

**Figure 10:**
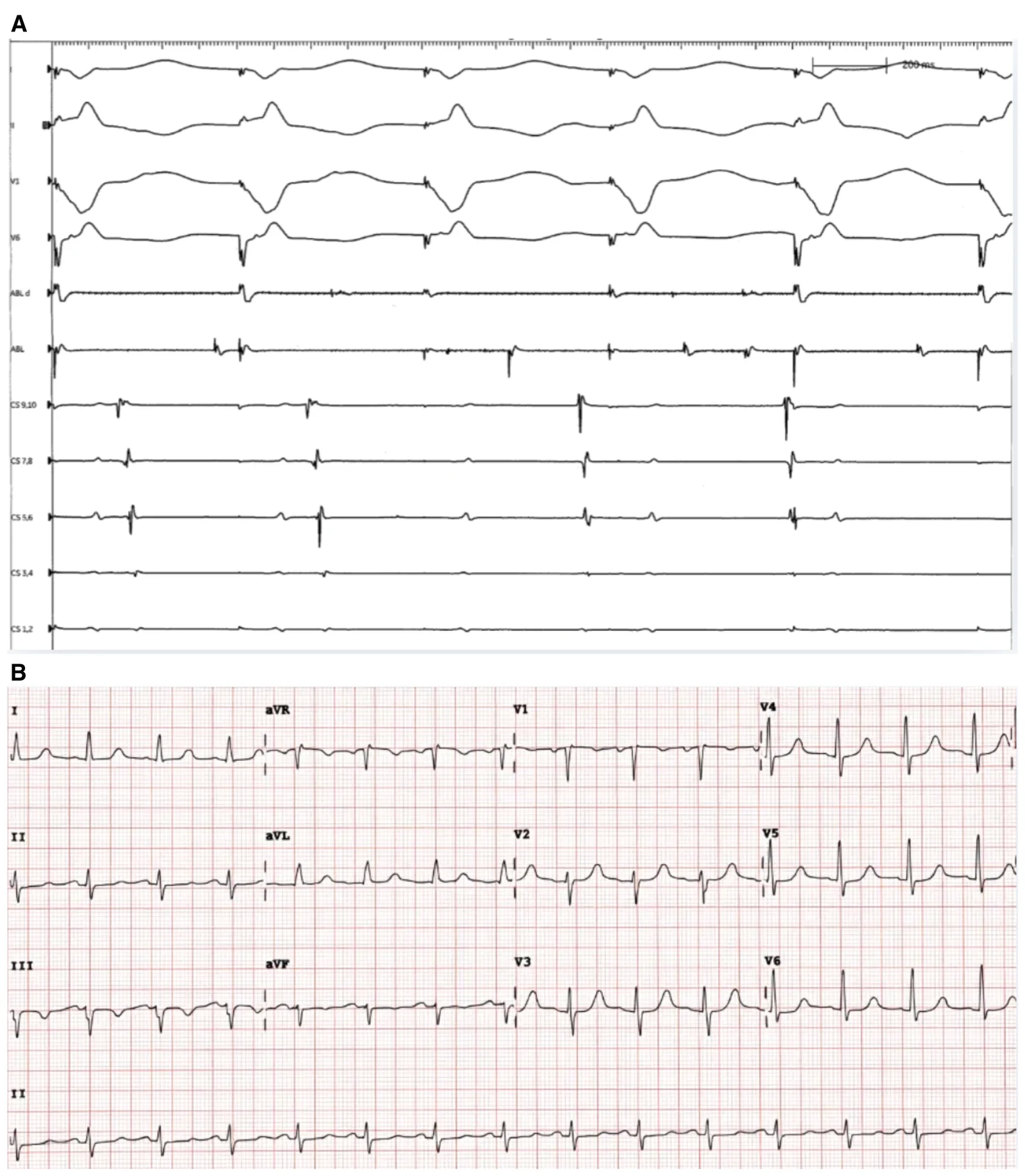
**A:** Ventricular pacing after administration of 18 mg of adenosine demonstrating loss of retrograde conduction over the accessory pathway. **B: **Post-ablation electrocardiogram demonstrating loss of pre-excitation.

Institutional review board approval was not required for this single retrospective case report. Written informed consent for publication (including images/ECGs) was obtained from the patient. All principles of the Declaration of Helsinki were followed.

## Discussion

APs associated with the CS are extremely uncommon and considered a rare subset of posteroseptal or left posterior APs. These pathways are located epicardially with connections between the CS myocardial coat and the ventricular epicardium. CS diverticula are found in approximately 7%–11% of all patients with posteroseptal or left posterior APs.^1,2^ CS diverticula have been identified in 21% of more specific populations with coronary–ventricular accessory connections that have required CS venous ablation. In some larger retrospective studies, CS diverticula compose approximately 0.36% of all AP ablations.^3,4^

Evaluating ECGs can be a key component in identifying the AP origin associated with the CS. The classic ECG pattern of CS diverticulum-related posteroseptal APs is a prominent negative delta wave and QRS complexes in the inferior leads (II, III, and aVF). An immediate negative delta wave or QRS in lead II is very specific for suspecting this location, although negative delta waves in the inferior leads generally suggest a posteroseptal pathway. These ECG findings indicate that the initial ventricular activation is being directed superiorly and anteriorly from the posterior epicardial insertion site within or near the CS diverticulum. This pattern arises due to the pre-excitation that originates from the subepicardial posterobasal left ventricle, ultimately resulting in an inferiorly directed electrical signal that appears as a negative deflection on the ECG.^5,6^ Additionally, a steep delta wave in aVR (specificity, 98%; positive predictive value, 88%) and a deep S-wave in V6 (R ≤ S) are ECG findings highly supportive of a CS-related pathway, and a combination of these two features has up to 99% specificity.^6^ In this case, the peculiar finding of an immediate negative delta wave in lead II together with multiple failed ablations was highly suggestive of an epicardial CS-associated pathway, which prompted targeted mapping despite prior misclassification of posteroseptal endocardial AP.

These pathways are extremely difficult to ablate with standard endocardial approaches, which have high initial failure rates; nearly 50% of patients with CS diverticulum pathways have one to three prior unsuccessful ablation attempts.^4^ Standard right or left posteroseptal endocardial ablations are often ineffective due to the combination of an epicardial course, oblique fiber orientation, and anatomical complexity associated with thick muscle strands within the diverticulum neck or middle cardiac vein.^1,6^ The recurrence rate after ablation is high as 23% in the pediatric population; however, long-term procedural success is associated with appropriate ablation techniques.^7^

In common practice, it is recommended and preferred to perform a CS venography when a CS-related pathway is suspected based on ECG patterns or failed endocardial ablations as it helps delineate the diverticulum anatomy and guides ablation to the neck where AP fibers converge.^8,9^ This approach has led to improved success rates; however, the risk of venous perforation or coronary artery injury remains significant, with coronary artery injury reported in up to 9% of CS AP ablations in young patients.^7^ Although we were able to localize the pathway without the need for venography in our patient, we believe that, in many cases where the ablation or mapping catheters cannot reach the target, venography may still be needed for better visualization and target localization.

In our case, however, successful ablation was achieved without fluoroscopy or CS angiography, relying solely on 3D electroanatomic mapping (CARTO™ system) to reconstruct the RA, vena cava, and CS geometry. Early ventricular activation and fused AV signals within the CS, distal to the ostium, localized the target and allowed for immediate pathway elimination with a contact force–sensing ablation catheter. This fluoroless strategy helped minimize radiation exposure while also demonstrating that high-resolution 3D mapping can suffice for anatomical guidance and identification with no need for contrast venography. The use of 3D mapping in AP ablations has been associated with reduced fluoroscopy time without significant increases in procedure time.^10^

## Conclusion

Patients with prior multiple failed ablations, as in this case, underscore the importance of maintaining high suspicion for epicardial CS-related pathways when classic ECG patterns are present. To the best of our knowledge, this case represents the first reported successful ablation of a CS-associated epicardial AP performed entirely without fluoroscopy or CS venography. Though zero-fluoroscopy AP ablations are well established and 3D mapping has been used in CS diverticulum cases with venography, the combination of a fluoroless technique and an absence of CS venography represents a novel approach.^9,10^ This case illustrates the evolving role and use of advanced 3D mapping systems, which may be a safer alternative for patients with recurrent arrhythmias and challenging anatomy.
